# Low-dose proton induced genetic alteration in cingulate cortex and declined its relevant cognitive function in behaviors

**DOI:** 10.3389/fnbeh.2025.1514579

**Published:** 2025-03-10

**Authors:** Gyutae Kim, Hyelim Park, Kyu-Sung Kim

**Affiliations:** ^1^Research Institute for Aerospace Medicine, Inha University, Incheon, Republic of Korea; ^2^Department of Otolaryngology Head and Neck Surgery, Inha University Hospital, Incheon, Republic of Korea

**Keywords:** cognition, proton, gene expression, cognitive behaviors, space radiation

## Abstract

Environmental radiation poses health risks to the central nervous system (CNS) as well as the internal organs. While the technology for managing radiation has improved, the effects of low-dose radiation in the long term are still considered as a health-related risky factor. The clinical and space radiation studies suggested cognitive threat from proton, but the inconsistent behavioral responses to low-dose proton made their cognitive effects elusive. Here, we examined the low-dose proton-induced functional changes by measuring genetic and behavioral responses. Total 54 mice (C57BL/6, 7 weeks, males) were used for this study. The genetic effects were tested using the brain tissue (cingulate cortex, CC), one of core regions for cognition, and the behavioral responses were evaluated by open field (OFT) and radial maze tests (RMT). In 4 weeks after irradiation, all genes (HSPA, GFAP, MBP, NEFL, NEFM) showed peak inflammatory responses (*p* < 2.05×10^−3^), and these reactions were resolved in 3 months, returning to the initial level of foldchanges. The behavioral changes were identified between 4 weeks and 3 months, which was after the peak genetic inflammatory period. The moving distance and the speed were maintained up to 4 weeks, but both motional factors decreased with significance after 4 weeks (*p* < 0.126×10^−3^). Unlike the results in OFT, no parameters in RMT showed a significant difference among the groups. Considering the overall results, low-dose protons induced reversible genetic alteration in the central regions over time, and their delayed effects on cognitive behaviors were limited, with consequences varying depending on the functional types of cognition. Our current findings are expected to provide critical information for the development of substantive regulations for astronauts’ health and clinical use of proton.

## Introduction

Current environmental hazards have increased human health threats, and the radiation ranged from natural sources to human activities such as clinical equipment plays a significant role in accumulating health risks (Karuppasamy et al., 2024). Beyond the immediate physical effects on the skin and bones, radiation’s indirect impact on internal organs like the heart, the liver, the thyroid gland as well as generative organs is becoming more pronounced (Belzile-Dugas and Elsenberg, 2021; Ogilvy-Stuart and Shalet, 1993; Reiners et al., 2020; Zhu et al., 2021). Particularly concerning is the central nervous system (CNS), which is highly sensitive to radiation (Katsura et al., 2021; Kovalchuk et al., 2017; Ma et al., 2024). The effects of radiation exposure on CNS are significant, as they directly impact an individual’s quality of life by altering essential functions. Extensive research and technological advancements in radiation studies have led to the development of defense mechanisms and technical approaches against high-dose radiation exposure, and ongoing research aims to enhance the effectiveness of these technologies (Mohania et al., 2017; Porcerelli et al., 2017). However, research into the biological effects of long-term and low-dose radiation exposure is still in its early stages, and the standards remain inadequately defined (Elsner et al., 2023; Janiak et al., 2023; Khan et al., 2021; Mahesh et al., 2023; Shin et al., 2020). Accordingly, the comprehension of low-dose irradiated effects on the function of CNS are limited, and its overall relation from the genetic to the behavioral responses is loosely understood.

Proton, which is the dominant particle in space radiation, has been mainly used for a clinical purpose, specifically cancer therapy (Lamirault et al., 2020; Pulsifer et al., 2015; Tang et al., 2022), but its excessive exposure is known to cause cognitive impairment (Bellone et al., 2015; Kantarci, 2013; Pulsifer et al., 2015; Tang et al., 2022; Williams et al., 2022). Previous studies addressed possible proton-induced functional decline, such as neurochemistry (Belov et al., 2019; Shukitt-Hale et al., 2004), neurophysiology (Bellone et al., 2015) and functional images (Suckert et al., 2021; Tang et al., 2022). Clinical studies have also reported cognitive decline following proton therapy with a high-powered energy, initiating the functional changes in CNS (Lamirault et al., 2020; Sienna et al., 2024). While a high-dose proton therapy effectively shields non-target areas, it has the limitation in mitigating the incidental low-dose irradiated effects on CNS.

As distinct from the proton effects by the clinical uses, the cognitive decline by radiation is also an essential topic for advancing the field of space exploration. Unlike Earth, the cosmic environment continuously provides space radiation, composed of the trapped particles from the Earth’s magnetic field, solar flares, and galactic cosmic rays with high-energy protons and heavy ions (Papadopoulos et al., 2023). Although the shielding technology has effectively blocked the high-dose space radiation, space exploration programs are still threatening the astronauts’ health by low-dose space radiation due to the expanding duration in space (Dynan et al., 2022; Wakayama et al., 2021). While the accumulated data on the low-dose radiation-induced effects on cognitive function are still lacking, various studies have insisted on the cognitive impacts by high-energy and low-dose ionizing radiation (Betlazar et al., 2016; Pasqual et al., 2021). Accordingly, the low-dose (<100 cGy) ionizing radiation is known to damage the brain blood vessels by altering gene expression, which can lead to neuronal defects (Lowe et al., 2009). Also, studies reported the decrease of the ratio between cAMP and cGMP as well as cAMP level, which suggested the failure of neurophysiological function and the generation of various diseases and indicated the cognitive and learning decline through the damages to crucial proteins (Batty et al., 2017; Cho et al., 2014). These findings have supported that the functional deficits occurred across various levels due to the low-dose radiation, contributing to the functional decline in cognition following exposure (Acharya et al., 2015; Pasqual et al., 2020; Pasqual et al., 2021). Contrarily, despite their prevalence in cosmic radiation and clinical applications, the low-dose proton-induced effects on the function of CNS remain controversial, demanding the overall examination from neurogenetics to cognition-related behaviors (Cucinotta et al., 2019; Sorokina et al., 2021).

Here, we aimed to investigate the impact of low-dose proton on cognition, building upon the insights gained from previous ionizing radiation studies. Specifically, we explored the relation between the changes in neurogenetic and cognitive behavioral responses which were altered by proton irradiation. For this, some behavioral experiments (open field & radial maze tests) were performed using animals (SD rats), and their regional tissues from cingulate cortex, closely related with cognitive function, were genetically tested based on quantitative real-time reverse transcription polymerase chain reaction (qRT-PCR). Previous studies using ionizing radiation have suggested that the radiation-induced functional decline in behavioral outcomes was caused by a series of reactions from genetic alteration and the impaired neural function by neuronal defects. Thus, no radiation-induced dysfunction is manifested immediately but rather unfolded over time. To assess the notion, the time delay of the behavioral outcomes following the genetic alteration was also examined in this study.

## Materials and methods

Fifty-four mice (C57BL/6, 7 weeks, males) were used, which were purchased from Orient Bio (KyungGi-do, Korea). Half of the total population (27 mice) was used for behavioral tests, and the other 27 animals were for a genetic test. Except during the experiments, all animals were housed in a facility with stable temperature (22–25°C), humidity (40–60%), ventilation (10–15 times/h), static pressure difference (>5mmAq), noise level (<60 dB) and a 12:12 h light–dark cycle.

### Animal preparation and proton exposure

**Figure 1:**
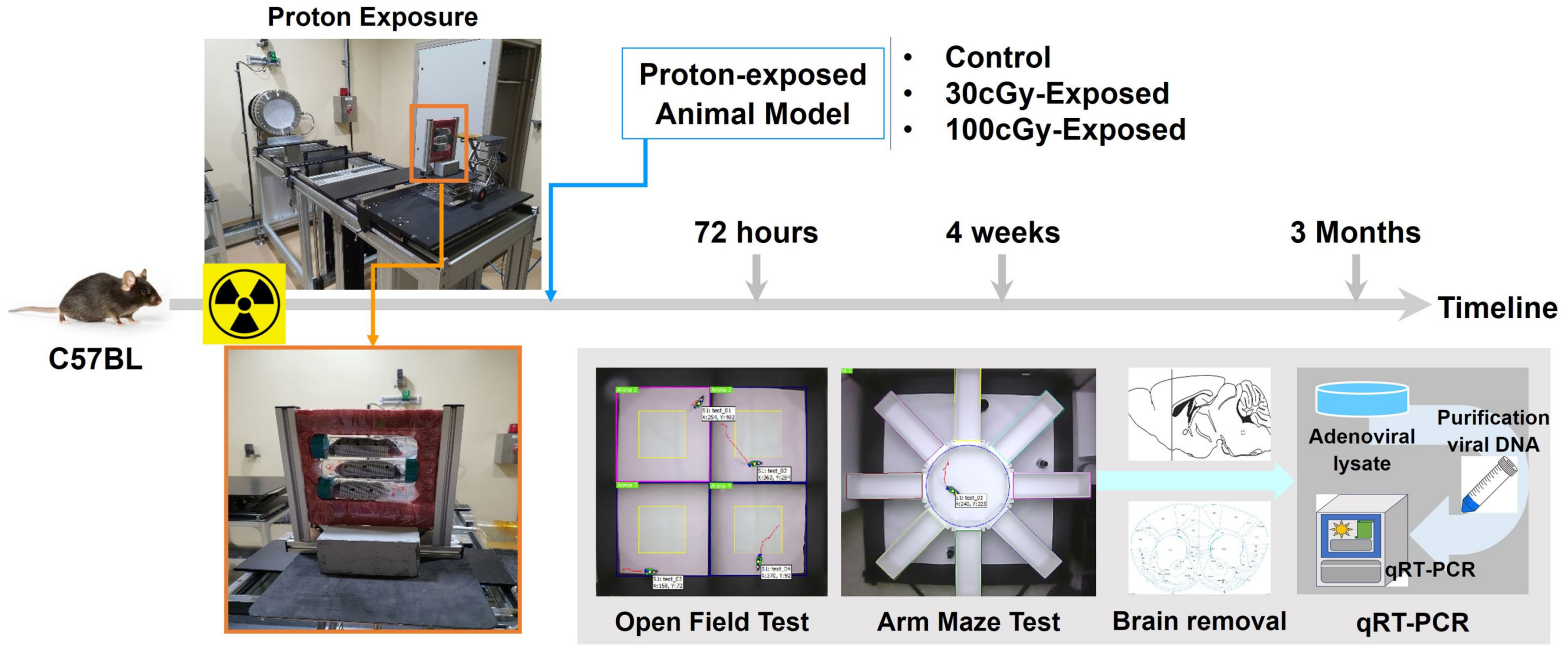
Experimental schematics. Relocated animals were irradiated with a single exposure to proton, and 3 groups were formed depending on the irradiated dose: control, 30 cGy-, and 100 cGy-exposed groups. The animals then underwent behavioral tests at specified periods (72 h, 4 weeks, and 3 months) after irradiation. After completing the behavioral tests, the animals were sacrificed, and the targeted brain tissue was removed for genetic evaluation by qRT-PCR.

Animals were grouped depending on the total dose (control, 30 cGy, and 100 cGy), and the proton irradiation was conducted at Korea Multi-purpose Accelerator Complex (KOMAC, KyungJu, South Korea) (Figure 1). Arriving at KOMAC, each animal was put into a falcon tube (50 mL) which had eight to nine small holes (diameter < 1 cm) after a 1-h break, and the limited space of the tube provided the animal’s positional stability during proton irradiation. Using a holding frame, 3 animals were exposed to every beam trial, and the prepared animals were positioned at about 1.5 m away from the beam outlet. Proton irradiation was conducted on a single exposure basis. The beam energy was 100 MeV, and its valid area was 10 × 10 cm^2^ (uniformity: 95.93%). The beam was delivered with 2.5% error rate, and these total doses were completed by the repeated beam pulses (energy per pulse: 0.0026–0.0035Gy/pulse).

To assess the stress by the beam exposure and the long transportation, the weight changes and the skin damage were examined before and after the relocations and the beam exposure. Skin injury was often reported after some proton therapies, and any abnormal skin deformans might indicate the effects by proton irradiation. At each sacrificing period (72 h, 4 weeks, and 3 months), the skins of animals were observed, and their conditions were scored based on some previous scoring systems (Jourdana et al., 2012; Pisciotta et al., 2020). The body weight was also measured at the same periods of time, and its changing rate was calculated for the percentile of weight difference to quantify the weight changes over the given period.

### Open field test

The open field (OPF) test, also known as fear/anxiety-like behavior test, was designed for the quantitative evaluation of animal’s locomotor activity as well as the animals’ moving pattern. All animals performed OPF at the given periods (72-h, 4-week, and 3-month after irradiation), and the parameters (moving distance and mean speed) were compared among the groups (Figure 1). For 10 min, each animal was allowed to move freely in a squared area (50 × 50 cm^2^), and all movements of four animals were simultaneously recorded with automated detection by a video tracking system (SMART 3.0, Harvard/PANLab, US). The analysis was performed off-line, and the parameters were separately obtained in a user’s defined imaginary (Center, 25 × 25 cm^2^) and the boundary zone (Edge). The moving distance was the total moving length in the zones, and the mean speed was the average moving speed with no resting basis. The measured parameters were presented by the means and standard deviation (STD) at the periods.

### 8-arm radial maze test

**Figure 2:**
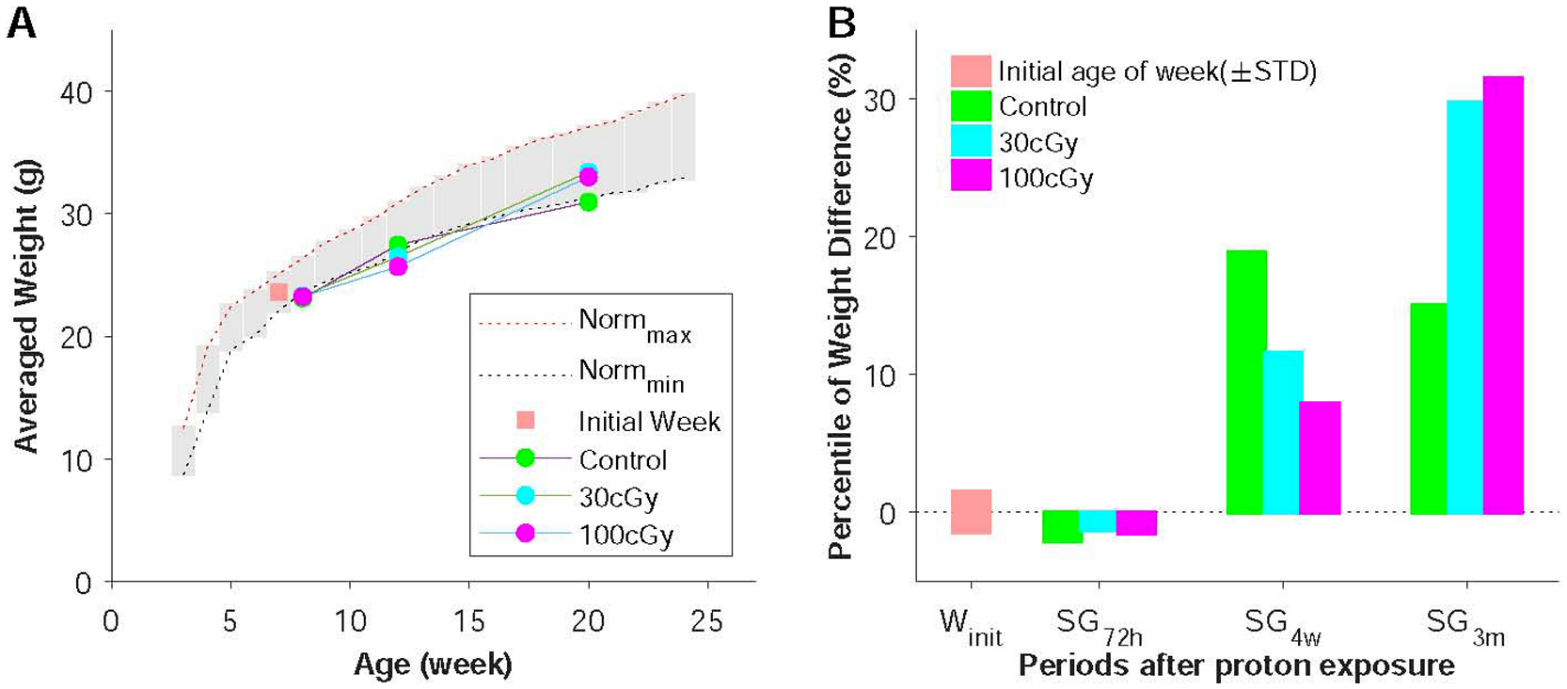
Weight changes before and after proton irradiation. **(A)** Age-related weights change in weeks. All animals were initially 7 weeks old (orange), and the measurement was conducted at 72 h (sub-group 72 h, SG72h), 4 weeks (SG4w), and 3 months (SG3m) after irradiation in control, 30 cGy-, and 100 cGy-exposed groups. **(B)** Percentiles of weight changes in the groups, which illustrated how much weight changed over the given period.

The maze test was conducted in a customized structure, which had a circular area (middle zone, 38 cm-diameter) and 8 rectangular shaped arms (50cmx10cm for each) (Figures 1, 2). All parts of the structure were individually covered with fine wire meshes. Owing to the mesh, the animal under the test session was forced to stay in the structure as well as the camera obtained the animal’s movements. The test lasted for 10 min, initially releasing the animals in the middle zone, and all animals underwent the test at the given periods as did in OPF. In the analysis, the number of entries to each arm was mainly counted, and the relative rate of entry was calculated, compared to the highest entering number to an arm during the test.

### Brain tissue and qRT-PCR analysis

Cingulate cortex (CC) is known as one of core cognition-related brain areas, and has multiple interconnections to other cognitive areas, such as amygdala, lateral prefrontal cortex, parietal cortex, and hippocampus. Due to its wide range to cover the cognitive functions, CC, specifically area 1 and 2 of Cingulate Cortex (Cg1 & Cg2), was chosen to assess the radiation effects at the genetic level. Generally, the location was ranged ±1 mm laterally, 1.5–2.0 mm ventrally, and its sagittal length was approximately 1.88 mm (range: 1.42 mm anterior & 0.46 mm posterior) based on the Bregma. Sacrificing animals was conducted by exposing to CO_2_. Each animal was placed in a transparent box, and CO_2_ was injected into the sealed space. After confirming the animal’s death, its CC was collected for quantitative real-time reverse transcription polymerase chain reaction (qRT-PCR).

The gene expression by qRT-PCR was initiated by RNA extraction from the tissues of CC in Qiagen RNeasy Plus Universal (Qiagen, Manchester, UK). The tissues (approx. 50 mg) were homogenized and loaded onto a purification column after mixing to ethanol. Then, the column was washed for its high purity. The amount of isolated RNA was subsequently determined by measuring absorbance at 260 nm using a Nanodrop spectrophotometer ND-1000 (Thermo Fisher Scientific, Waltham, MA. USA). For cDNA synthesis, RNA (300 ng) was used with High-Capacity cDNA Reverse Transcription Kit (Thermo Fisher Scientific, Waltham, MA. USA), and its PCR was amplified by AriaMx Real-time PCR System (Agilent Technologies, *Santa Clara,* USA). mRNA expressions were performed using AriaMx Real-time PCR System (Agilent Technologies, *Santa Clara*, USA) and qPCR Brilliant SYBR Master Mix (Agilent Technologies, *Santa Clara*, USA). Diluted cDNA (5 μL) was amplified with 1 μL of 0.2 μM primers, 10 μL of SYBR Green Master Mix, and 4 μL of nuclease-free water in a total volume of 20 μL. All Primers were purchased in Qiagen Primer (Düsseldorf, Germany); NEFL, HSPA2, NEFM, MBP, GFAP, and Gapdh. The relative gene expression levels were quantified using the comparative CT (2^−ΔΔCT^) method using AriaMx software and normalized to Gapdh. The amplification protocol was as follows: initial melting step at 95°C for 3 min, followed by 40 cycles of a 95°C melting step for 10 s, a 60°C annealing and elongation step for 15 s. After amplification, a dissociation curve analysis was performed to confirm the purity of PCR products. Cycling parameters for melting curve analysis were 30 s at 95°C, decreasing to 65°C, then increasing from 65°C to 95°C with a default rate of 0.5°C/s.

### Statistics

Tests for the alterations in the behaviors and the genetic foldchange among the different groups in the given periods were compared by 2-way ANOVA (significance level: 0.05), and the post-hoc tests were performed using Turkey-Kramer test. Behavioral and genetic results were compared based on the proton doses (0, 30, and 100 cGy) and the post-irradiation periods (72 h, 4 weeks, and 3 months). Tests focused on the core parameters in the examined behavioral and genetic responses as well as weights. In OFT, the moving distance and the mean speed were examined, and the number of entries to the arms was tested in RMT. The foldchange in genetic expression was also examined based on this given test.

## Results

**Figure 3:**
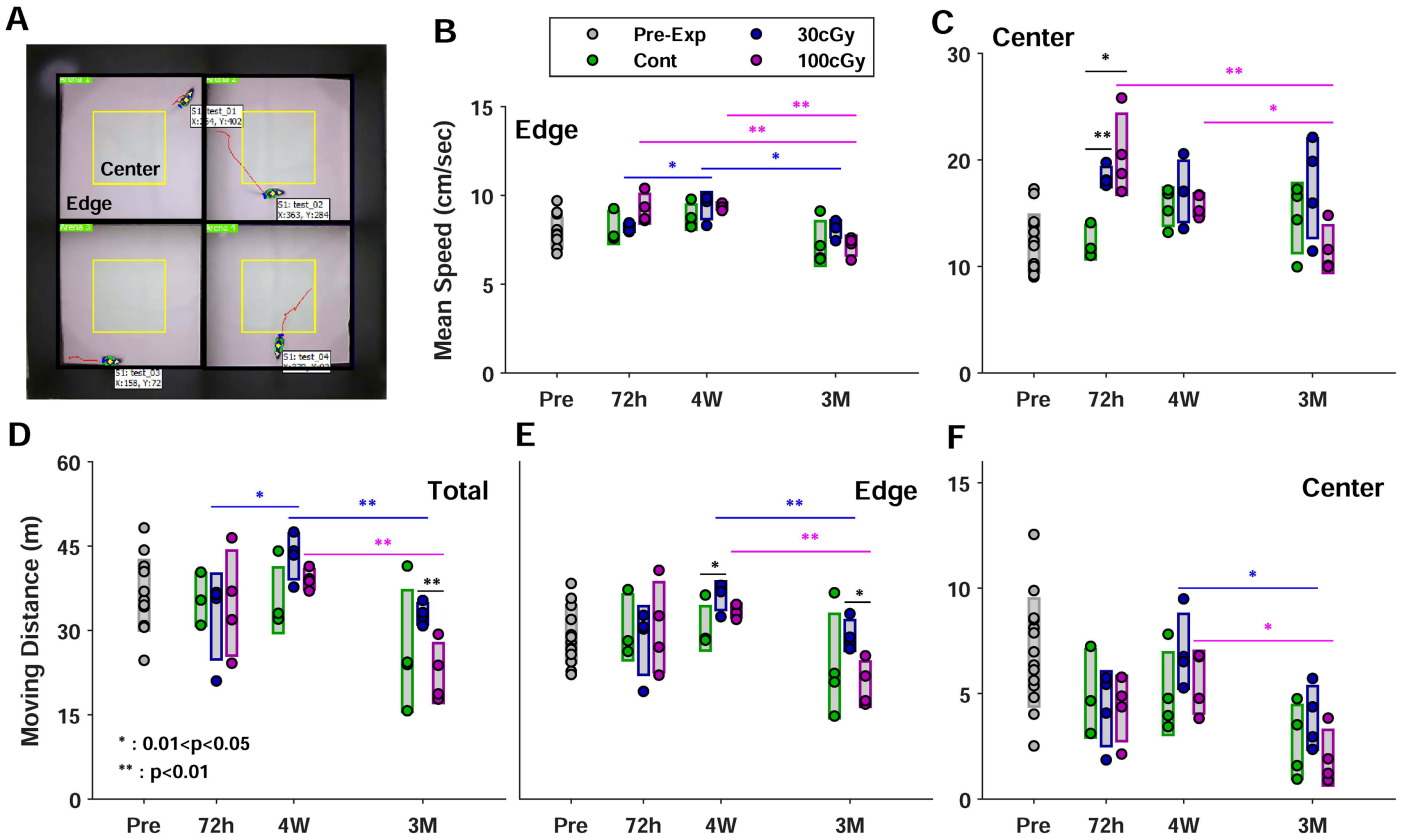
Open field test. **(A)** Top view of experimental setup for 4 animals. Each space was divided into an imaginary center (in yellow) and an edge. **(B)** Mean speed in the edge depending on the periods before and after proton exposure. Each group was represented in different colors; green for control, blue for 30 cGy-exposed, and purple for 100 cGy-exposed groups, and the circles and the bars indicated raw data and the relevant standard deviation, respectively. The same formats were applied for all the subplots. **(C)** Mean speed in the center **(C)** Total moving distance **(E)** Moving distance in the edge **(F)** Moving distance in the center. (*, 0.01 < *p* < 0.05; **, *p* < 0.01).

As designed, the grouped animals (control, 30 cGy-, and 100 cGy-exposed) underwent two behavioral tests and qRT-PCR in the given periods of time (72 h, 4 weeks, and 3 months) after the irradiation (Figure 1). At the time of examination, animals’ skin and their body weights were inspected to identify any physical damages by the radiation or the long-distance relocation. Based on the observation throughout the experimental process, no skin injury was found (0 score), indicating the dose (30 & 100 cGy) of proton caused no direct damage to the skin. On the other hand, the weights initially decreased and recovered as time proceeded (Figure 3). The weights in all groups (control, 30 cGy-, and 100 cGy-exposed groups) decreased at the period of 72 h, but they recovered with different rates depending on the dose of the exposed proton (18.90, 13.69, and 10.49% in control, 30 cGy-, and 100 cGy-exposed groups, respectively). Interestingly, the order in recovering rates of weights was inversed at 3 months as 12.74, 26.13, and 28.60% in control, 30 cGy-, and 100 cGy-exposed groups, respectively. The comparison of pair groups indicated all weights were periodically dependent (*p* < 2.05×10^−9^). The interaction of two factors (dose & post-period) was also significant (*F*(4,45) = 5.499; *p* = 0.001), suggesting both factors affected the weight changes (Figure 3A). The percentile weight changes of 3 sub-groups (SG_72h_, SG_4w_, and SG_3M_) indicated the long-distance relocation led to the initial weight reduction, and the irradiated effects in weight occurred from 72 h to 4 weeks (Figure 3B).

**Figure 4:**
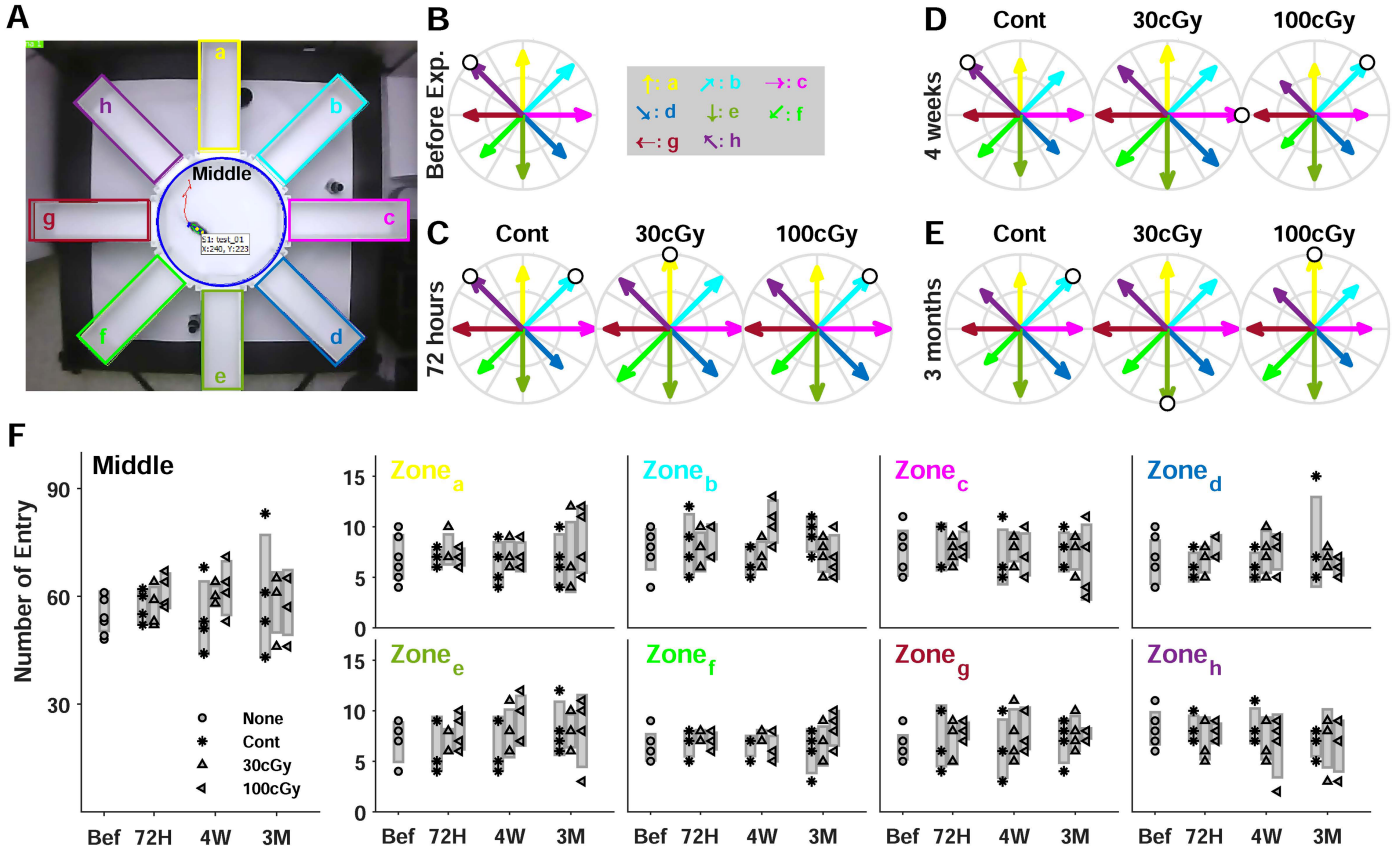
8-Arm maze test. **(A)** Top view of experimental setup. Each arm was labeled by alphabets (a-h in clockwise direction). **(B)** Relative rate of entry to the arms before proton exposure. The rate was calculated by dividing the entry numbers by the highest entry number (white circle). **(C)** Relative rate of entry at 72 h **(D)** Relative rate of entry at 4 weeks **(E)** Relative rate of entry at 3 months **(F)** Comparisons of entry numbers in each zone including the middle zone depending on the period after proton exposure.

In the open field test (OFT), the location of all animals was continuously monitored, and two parameters, moving distance and mean speed, were separately measured in both areas of center (inside of yellow square) and edge (outside of yellow square) (Figure 4A). In the same periods, both parameters were rarely different among the groups (*p* > 0.079) except the mean speed in the center at 72 h (*p* < 0.002). Most pair comparison in OFT indicated the behavioral alteration depended on the post-period of irradiation. The mean speed (cm/s) significantly decreased after 4 weeks in 30 cGy- and 100 cGy-exposed groups (*p* < 0.018) while the control group showed no difference in both zones (*p* > 0.495) (Figures 4B,C). In the edge, the mean speed of both 30 cGy- and 100 cGy-exposed groups decreased after 4 weeks, but that of 100 cGy-exposed group showed a more dramatic decrease between 4 weeks and 3 months (*p* = 1.52×10^−7^) (Figure 4B). At center, on the other hand, the difference in the mean speed was observed between control and 30 cGy-exposed group (*p* = 0.002), but other pairs of groups showed no significance (*p* > 0.079) (Figure 4C). From the periodic aspect, no significance in 72 h and 4 weeks was found (*p* = 0.496) while other pairs (72 h vs. 3 months & 4 weeks vs. 3 months) showed significant difference (*p* < 0.018), which indicated the irradiation affected the motional activities after a certain time of time. Nevertheless, the interaction of 2 factors in mean speed was significant (*F*(4,45) = 3.13; *p* < 0.024). In the moving distance, the periodic alteration was continuously maintained. The moving distance in total and center changed in 4 weeks and 3 months (*p* < 0.049) (Figures 4D,F), but that in edge had no alteration up to 4 weeks (*p* = 0.122) (Figure 4E). On the other hand, ANOVA test identified the periods after irradiation rarely affected the alteration in mean speed and moving distance (*F*(2,45) = 19.6; *p* > 0.1532) except the mean speed in center (*F*(2,45) = 6.84; *p* = 0.0025). Instead, the proton dose was key factor to induce the functional alteration in both mean speed and moving distance (*F*(2,45) = 4.12; *p* < 0.0227). In addition, no interaction between 2 factors was observed.

The radial maze test was mainly analyzed by counting the number of entries to each arm (Figure 2A). Using the maximum number of entries in each trial, the relative rate of entry was calculated (white circle), but the maximum number showed no directional preference (Figures 2B–E). Also, the rates to the arms had no constant changes depending on the proton dose. The relative rate of entry before the irradiation ranged from 0.625 to 1, and the control groups at 72 h, 4 weeks and 3 months also a similar distribution compared to that of pre-exposure. Moreover, the distribution of rates at 3 months was similar no matter how much an animal was exposed to proton (Figure 2E). However, the animals’ entrance into the arms at 4 weeks indicated the rates were unequally distributed, resulting in significant differences among the groups. Especially, the entering rates of 100 cGy-exposed group were different from those of 30 cGy-exposed group (*p* = 0.0013), and it suggested that an abnormal moving pattern was identified at 4 weeks after the proton irradiation (Figure 2D). The comparison in each zone (a-h) indicated the entrance to a specific zone was independent of the proton dose and the period of irradiation (Figure 2F). Most entering numbers to the zones as well as the middle area showed no significant difference at the given periods after irradiation (*p* > 0.262), and no irradiated dose also made any difference (*p* > 0.098). As shown in both the entering numbers and its rates to the zones, the results in the maze test suggested the alteration of moving pattern based on the multiple choices to a pathway occurred with the dose of the irradiated proton (100 cGy) at a specific period (4 weeks) after irradiation. The analysis based on ANOVA also showed no significance in the number of entry to different arms regarding to neither proton dose (*F*(2,45) = 1.28; *p* > 0.2881) nor post-irradiation period (*F*(2,45) = 2.83; *p* > 0.0697), which implied that no animal’s instinct to explore new places was affected by given protons. Also, no interaction of 2 factors was identified (*F*(4,45) = 2.56; *p* > 0.051), except the entry to b zone (*F*(4,45) = 3.1; *p* = 0.024).

**Figure 5:**
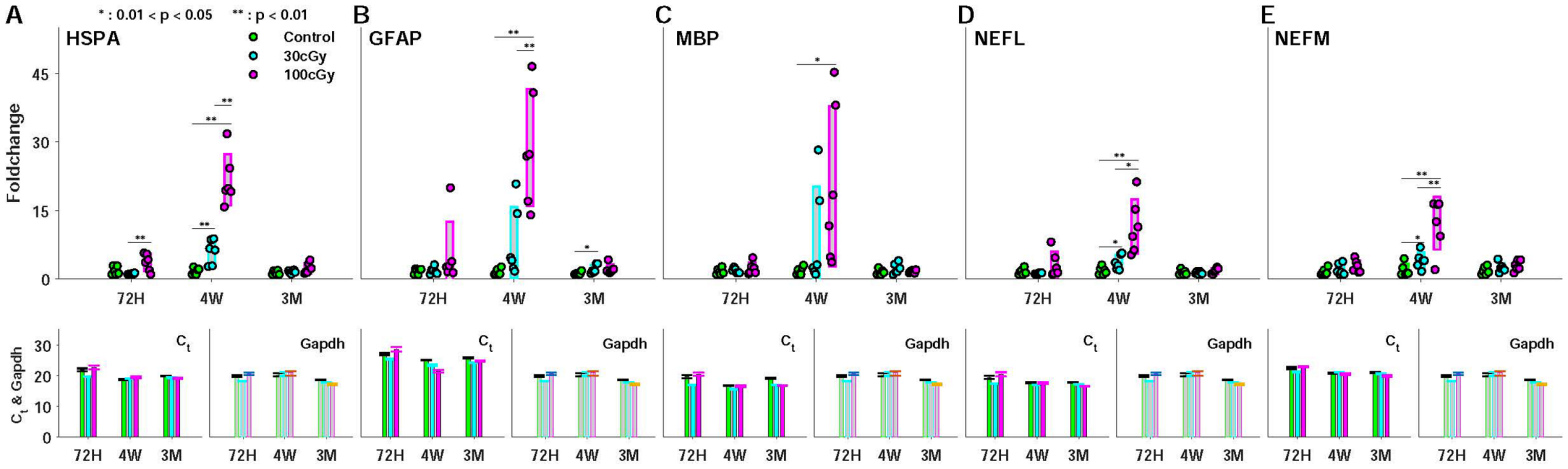
Expression (foldchange) of 5 genes (HSPA, GFAP, MBP, NEFL, and NEFM) during the periods (72 hours, 4 weeks, and 3 months) after proton irradiation. Each representation was composed of 3 subplots for foldchange (left), cycle threshold (Ct) (middle), and Gapdh (right). **(A)** heat shock protein (HSPA); **(B)** Glial Fibrillary Acidic Protein (GFAP); **(C)** Myelin basic protein (MBP); **(D)** Neuro- filament light (NEFL); **(E)** Neurofilament medium (NEFM).

The genetic responses to proton were examined by the foldchange of 5 genes (HSPA, GFAP, MBP, NEFL, NEFM) at the given periods, considering their neuronal functions (Figure 5). For instance, heat shock proteins (HSPs) play a role in protecting cells and systems from external stresses, so-called neuroprotector. Especially, HSP70 (HSP with 70 kDa molecular weight) supports forming memory in the brain, and its specialized role relates to the age-related function. GFAP manages various central processes, such as neural communication and the function of brain blood barrier (BBB). This gene is also known as an early marker for apoptosis in cognition-related brain regions, such as hippocampus and ACC. The other genes (NEFL, NEFM, MBP) are involved in myelination. Based on the roles in neural function, the genes were selected, and the foldchange was mainly examined to assess the effect of the low-dose proton. Due to their specialized functions for neuronal activity, the genetic alteration implied neural dysfunction (see Brain Tissue and qRT-PCR Analysis). Using the tissue from the cingulate cortex (CC), the cycle thresholds (C_t_) and the Gapdh were measured, and the foldchange was calculated to estimate the proton-induced effects. In all genetic responses, the dose-dependent significance was found between control and 100 cGy-exposed group (*p* < 0.021), and the comparison between 30 cGy- and 100 cGy-exposed groups also resulted in statistic difference (*p* < 4.50×10^−4^). However, that of MBP indicated that there was no significance between 30 cGy- and 100 cGy-exposed groups (*p* = 0.252). On the other hand, the periodic significance maintained the same consequence, demonstrating the proton irradiation affected the genetic responses by increasing the foldchanges only at 4 weeks after the irradiation (*p* < 9.57×10^−10^, *p* < 1.15×10^−5^, *p* < 2.05×10^−3^, *p* < 6.12×10^−5^, and *p* < 3.50×10^−5^, for HSPA, GFAP, MBP, NEFL, and NEFM, respectively) while no significance between 72 h and 3 months (*p* > 0.818) (Figures 5A–E). ANOVA test noted that additional information suggested both factors, such as proton dose and post-irradiation time, were critical sources to show the proton-induced effects on the genetic alteration (*F*(2,45) = 8.89; *p* < 0.0006 and *F*(2,45) = 3.9; *p* < 0.0273, respectively). In all genetic responses, 2 factors (dose & post-period) showed significant interactions in causing the responding alteration (*F*(4,45) = 3.45; *p* < 0.015).

## Discussion

The expansion of space exploration and the growing clinical use of radiation have led to increased interest in the deterministic effects of radiation, which requires the establishment of the long-term safety standard for low-dose radiation (Ali et al., 2020; Mahesh et al., 2023; Zhou et al., 2023). Particularly, it has been reported that clinical radiotherapy using protons as well as other types of radiation can cause cognitive impairment even at lower doses than the required for treatment (>25Gy) (Lamirault et al., 2020; Mash et al., 2023). Unlike genetic and molecular effects, however, the cognitive effects on behaviors are still controversial (Bekal et al., 2021; Pasqual et al., 2021; Shin et al., 2020). The inconsistent results in behaviors overlooked the cognitive effects by radiation, and it disturbs the establishment of the long-term regulation under the low-dose radiation. In this study, we attempted to explore the relation between the genetic and behavioral responses to low dose proton, and we attempted to explain the alteration of cognitive behaviors initiated from the genetic alteration. Through their quantifications, the functional changes by low-dose proton were elucidated, which has been considered as a weaker source than other ionizing radiation in causing any functional decline (Jumaniyazova et al., 2023; Zebrev et al., 2024). To justify the low-dose proton-induced cognitive effects, we measured both genetic and behavioral responses using the irradiated animals. Our results indicated proton caused the genetic alteration in the central region (cingulate cortex, CC), and the relevant effects peaked at 4 weeks after irradiation (Figure 5). As noted, the targeted genes were essential for the normal functioning of myelination, neuroprotection, neural communication, and blood–brain barrier functions, and their changes implied the neural dysfunction by proton, eventually causing the behavioral abnormality (Isles, 2015; Mc Mahon et al., 2003; Yao et al., 2014). On the other hand, the genetic alteration caused by the low-dose protons appeared with a time delay. In all genetic responses, no statistical differences were found between control and 30 cGy-exposed group (*p* > 0.203), but the genetic alteration was evident in 4 weeks (*p* < 2.05×10^−3^). The genetic responses were resolved in 3 months, and similar responding patterns were observed in all genes used in this study. These results suggested the low-dose proton-induced genetic alteration recovered over time after reaching the peak responding point (Figure 5). Interestingly, a similar change was identified in the percentile of weight difference (Figure 3B). At 4 weeks, the 100 cGy-irradiated group gained less weight than other groups, but the gaining rate in weight was reversed at 3 months. These sequential changes in genes and weights demonstrated the genetic alterations were related with the weight reduction, agreeing with previous obesity studies (Chatterjee et al., 2021; Lamiquiz-Moneo et al., 2019).

Considering the proton-induced genetic responses over time, the behavioral pattern altered with additional time delay. During the 3 months, the total moving distance of the irradiated groups significantly decreased while no change was observed in the control group (Figure 4D). Especially, the total moving distance was maintained up to 4 weeks, and most animals tended to decrease their moving distance after 4 weeks of peak genetic inflammatory responses. Based on the serial process from genes to behavior, the delayed outcomes of behaviors were reasonable after the genetic changes. Our rationale was that the genetic alteration caused neurocognitive dysfunction inducing the altered behavioral patterns with time delay. Along with this reasoning, the time delays before the genetic and the behavioral impairments were anticipated, and our results supported the rationale suggesting that the low-dose proton reduced cognitive performance increasing the genetic alteration as the accumulated data demonstrated (Daugherty et al., 2019; Khacho et al., 2017; Ropers, 2010; Slaney et al., 2023; Spoto et al., 2024). The moving speed also showed the delayed functional decline. The mean speed in both edge and center decreased 3 months after irradiation, and no reduction was identified before this period (Figures 4B,C). Thus, the behavioral alteration in OFT indicated the functional decline by low-dose proton irradiation. The dose-dependent effects were also supported by ANOVA test, indicating the proton-induced alteration of the parameters in OFT was mainly caused by the increased dose. Even though local significance was identified based on periods after irradiation, the additional test (ANOVA) suggested that the behavioral changes as time were insignificant.

On the other hand, the 8-arm radial maze test (RMT) identified no behavioral abnormality (Figure 2). An even distribution was examined by counting the entering number to the different zones (center & zone a-h), which was based on the animal’s instinct to explore new places (Mei et al., 2020; Penley et al., 2013), but the results suggested no significance depending on the irradiated dose and the lapse of time (*p* > 0.124). Noted that the general neural pathways for OFT and RMT originated from different central regions. The amygdala and mesolimbic areas provide psychomotor neural activity for OFT (Morel et al., 2022; Takita et al., 2020; Yawata et al., 2023), and the animal’s brain receives the neural information of memory and learning for RMT mainly from the hippocampus (Kim et al., 2018; Kohler et al., 2022). However, these regions are neuroanatomically connected to the cingulate cortex (CC), where the genetic evaluation was conducted in this study (see Brain Tissue and qRT-PCR Analysis). Structurally, CC in the limbic system is divided into anterior (ACC) and posterior CC (PCC). ACC involves goal-seeking behaviors such as reward-seeking and punishment avoidance and plays a role in the evaluation of goals based on behavioral outcomes (Kim et al., 2021; Rolls, 2019). In primates, including humans, ACC is known to receive information about emotions and rewards from the orbitofrontal cortex (OC) and amygdala while the behavioral and the memory neural information are sent from the parietal cortex (PC) and the hippocampus, respectively. On the other hand, in rodents such as mice, it is known that the anatomical separation of OC and PCC is still elusive, and it hardly defines their neural information (Foster et al., 2023; Xiang et al., 2023). Taken together, it was plausible to deduce that the proton-induced genetic alteration in CC affected the psychomotor responding patterns in OFT, but the low-dose proton rarely influenced the function of learning and memory implemented in RMT. As a result, the low-dose proton-induced effects on cognitive behaviors were limited despite the genetic alteration in the relevant central regions.

### Limitation

Current study selected 5 genetic candidates to represent the proton-induced damages, and it successfully provided the genetic damages, which were related with the functional alteration in inflammation or apoptosis. However, their responses were insufficient to explain the effects on the synaptic changes or relevant mitochondrial activities, which was known as the initial alteration after irradiation. The other limit was related to neural signal recordings in specific cognitive regions. Neural signals in a specific brain area could provide a possible functional link to cognitive behaviors, which were initiated by the relevant neural activity. However, the neural information was not assessed focusing on the fundamental aim of this study. Thus, few direct connectivity from the genetic to behavioral change was identified while the overall relation in the genetic and behavioral responses by low-dose proton was demonstrated.

## Conclusion

Radiation-induced functional changes in central nervous system (CNS) have been proposed through a variety of mechanisms from molecular biology to behavioral responses. In today’s era of space exploration as well as highly demanding use of clinical therapeutic purposes, the radiational effects should be understood in the aspect of not only a direct physiological response but also a multi-layered functional change such as cognitive function. Although the advanced technology in shielding radiation has eliminated some associated risks under high doses, the long-term effects of low-dose radiation on the central nervous system are still elusive. In this study, the effects of protons, which account for a significant portion of cosmic radiation, were examined based on genetic and behavioral responses related to cognitive functions. Unlike previous low-dose proton-induced effects on cognitive function, our results indicated that consistent cognition-related changes in behaviors as well as the relevant genetic alteration, occurring at a certain period (4 weeks) after proton exposure. Also, the cognitive changes in behaviors appeared after the genetic alteration with a time delay, suggesting that there was a functional interconnection between the genetic and the behavioral changes by low-dose protons. To demonstrate their systemic link, the future study requires uncovering the neural activities that connect the series from the genetic to the behavioral responses, and it would explain the impaired cognition caused by low-dose proton.

## Data Availability

The datasets presented in this study can be found in online repositories. The names of the repository/repositories and accession number(s) can be found in the article/supplementary material.
